# Structural Breaks in Mean Temperature over Agroclimatic Zones in India

**DOI:** 10.1155/2014/434325

**Published:** 2014-08-27

**Authors:** Ranjit Kumar Paul, P. S. Birthal, Ankit Khokhar

**Affiliations:** ^1^Indian Agricultural Statistics Research Institute, New Delhi 110012, India; ^2^National Centre for Agricultural Economics and Policy Research, New Delhi 110012, India

## Abstract

Amongst Asian countries India is one of the most vulnerable countries to climate change. During the past century, surface temperature in India has shown a significant increasing trend. In this paper, we have investigated behavior of mean monthly temperature during the period 1901–2001 over four agroclimatic zones of India and also tried to detect structural change in the temperature series. A structural break in the series has been observed at the national as well regional levels between 1970 and 1980. An analysis of trends before and after the structural break shows a significant increase in July temperature in the arid zone since 1972.

## 1. Introduction

Agriculture is more vulnerable to climate change than any other economic activity. Thus, the effects of climate change on agriculture and food supply are likely to be severe [1, 2]. Higher temperatures reduce yields of food crops while encouraging infestation of insect pests and diseases. According to the latest estimates, earth's linearly averaged surface temperature increased by 0.74°C during 1901–2005 [3]. A large number of studies on linear trend of temperature pattern are available whereas a few studies have focused on the change in pattern. Some studies on urbanization and related temperature variation indicate that the impact of urbanization on the mean surface temperature would be no more than 0.05°C per 100 years [4], while others have reported a rise in minimum temperature by 2–5°C per 100 years in several large cities [5]. It is understood that the observed warming is not only due to increased concentration of greenhouse gases but also due to urbanization [6]. The urbanization effect is pronounced for minimum temperature, and the magnitude of the effect is well correlated with the population of the city in which meteorological observatory is located [7].

During the past century, surface temperature over India has shown a significant increasing trend [8], confined mainly to maximum temperature. An investigation into long-term variation in seasonal and annual surface air temperature at six industrial and nonindustrial locations in India has shown that while there was no significant trend in temperature at nonindustrial locations, at the industrial locations there was either a cooling tendency or cessation of warming after the late 1950s [9]. In a study of the effect of urbanization on meteorological parameters in fifteen cities in India with population size more than one million it was found that the density of population, distance between tall buildings, vehicular population, and the industrial development have played an important role in controlling the urban climate [10]. Some of the urban locations in India are becoming increasingly vulnerable to extreme events in weather and climate [11]. In India 60–70% of meteorological stations in the coastal belt showed an increasing trend in critical extreme maximum day temperature and increase in night temperature during the summers [12]. Most studies conclude a rise in temperature. Some studies [9] reported cooling at industrial sites.

A large number of studies have analyzed temperature trend either at national level or at state level. Estimates at these levels are too aggregative and mask the regional variation in climate. India being a large country has considerable heterogeneity in agroclimatic conditions even within a state or subdivision. Thus, an analysis of climate variables at a more disaggregated level, say district, is desirable for a better understanding of their location-specific behavior. The study of temperature trends at zone level may provide inputs to policy makers to design regionally differentiated adaptation and mitigation strategies to cope with climate change.

Structural changes or “breaks” may affect key economic and financial time series such as output growth, inflation, exchange rates, interest rates, and stock returns. A small subset of the papers has reported evidence of breaks in economic and financial time series [13–21]. A key question that arises in the context of time-series forecasting is how future values of the variables of interest might be affected by breaks. Some studies view structural breaks as the main source of forecast failure [22, 23].

In this paper, we have examined trend in temperature during the period 1901–2001 at the level of an agroclimatic zone by clustering the districts having similar agroclimatic conditions into homogeneous zones and also identified structural breaks in the temperature series.

## 2. Methodology

India is a large country with considerable heterogeneity in climate. Thus, an analysis of climate behavior at aggregate level may mask its true behavior at disaggregated level, that is, states or districts. Many Indian states have a quite large geographical spread with varying climates. In this paper we analyzed behavior of temperature at the level of an agroecology which by and large is homogeneous in climate. Following the methodology of aggregation as in [24] we clustered districts of India into four broad agroclimatic zones, namely, arid, semiarid temperate, semiarid tropics, and humid. The semiarid tropics cover the largest geographical area, while the arid zone has the smallest geographical spread.

Data on mean monthly temperature from 1901 to 2002 was obtained from the Indian Meteorological Department (IMD) of the Government of India. We averaged monthly temperature of the districts in a zone for examining changes in it.

Following [25], the stability of trend line was tested initially by looking at the standard error of the estimate. CUSUM (cumulative sum) test [26] was used to detect the point of structural change and Chow test was performed to confirm the change. Then dummy variable technique, used in [25], was followed to test for the existence of a structural break in the series. For sake of brevity we report only the main results.

We tentatively select *T*_*b*_ as the year of the structural break. The break becomes effective one year after *T*_*b*_, that is, *T*_*b*_ + 1. Our null hypothesis here is that there is no significant break at *T*_*b*_ in the constant or trend. This is tested estimating the dummy variable regression of the following form:

(1) $$\begin{array}{c} y_{t}=\alpha_{1}+\alpha_{2}\cdot DVU_{t}+\beta_{1}\cdot t+\beta_{2}\cdot DVT_{t}+{\hat{\varepsilon }}_{t}, \end{array}$$

where *t* is a time trend, *DVU*_*t*_ = 0 if *t* ≤ *T*_*b*_ and 1 if *t* > *T*_*b*_, *DVT*_*t*_ = *t* × *DVU*_*t*_, and *ε*_*t*_ ~ *iid*(0, *σ*_*ε*_^2^).

From (1) we can estimate trend from the initial point of the series up to *T*_*b*_ as

(1a) $$\begin{array}{c} y_{t}=\alpha_{1}+\beta_{1}t+{\hat{\varepsilon }}_{t}. \end{array}$$

The trend from *T*_*b*_ + 1 to the end point of the series will be

(1b) $$\begin{array}{c} y_{t}=\left(\alpha_{1}+\alpha_{2}\right)+\left(\beta_{1}+\beta_{2}\right)t+{\hat{\varepsilon }}_{t}. \end{array}$$

Magnitude of change in parameters can be obtained by estimating the coefficients of *DVU*_*t*_ and *DVT*_*t*_.

We perform CUSUM test to detect the change points in the series that often suffers from structural changes. It is easy to understand and implement and can be used for testing and estimating the locations of the changes. The CUSUM test proposed in [27] is to test *H*_0_ : *σ*_*t*_^2^ is constant versus *H*_1_ : *σ*_*t*_^2^ is not constant over *X*_1_,…, *X*_*n*_, where {*X*_*t*_} is a series of independent random variables with (0, *σ*_*t*_^2^).

For the model *y*_*t*_ = *β*_*t*_ *x*_*t*_ + *ε*_*t*_, *t* = 1,2,…, *n*, the formal tests for structural stability of the regression coefficients can be computed from the standardized recursive residuals as

(2) $$\begin{array}{c} \omega_{t}=\frac{\vartheta_{t}}{\sqrt{f_{t}}}=\frac{y_{t}-\beta_{t-1}x_{t}}{\sqrt{f_{t}}},\\ f_{t}={\hat{\sigma }}^{2}\left[1+x_{t}{\left(X_{t}^{\prime }X_{t}\right)}^{-1}x_{t}\right]. \end{array}$$

The CUSUM statistic is defined as

(2a) $$\begin{array}{c} \text{CUSU}{\text{M}}_{t}=\overset{t}{\underset{j=k+1}{\sum }}\frac{{\hat{\omega }}_{j}}{{\hat{\sigma }}_{\omega }},\\ {\hat{\sigma }}_{\omega }^{2}=\frac{1}{n-k}\overset{n}{\underset{t=1}{\sum }}{\left(\omega_{t}-\overset{-}{\omega }\right)}^{2}. \end{array}$$

Under the null hypothesis that *β* is constant, CUSUM_*t*_ has mean zero and variance proportional to *t* − *k* − 1. CUSUM_*t*_ is also known as empirical fluctuation process (efp). Under the null hypothesis of no structural change, the limiting process for the efp is the standard Brownian motion. The efp reflects fluctuation in residuals and coefficients estimates. If the efp crosses the theoretical boundaries, the fluctuation is improbably large and then rejects the null hypothesis of no structural break.

CUSUM involves calculation of a cumulative sum which makes it “sequential.” Samples from a process *x*_*t*_ are assigned weights *ω*_*t*_ and summed as follows:

(2b) $$\begin{array}{c} S_{0}=0.\\ S_{t+1}=\max\left(0,S_{t}+x_{t}-\omega_{t}\right), \end{array}$$

When the value of *S* exceeds a certain threshold, then there is a change in value. *(2b)* detects changes in the positive direction only. When negative changes are to be found as well, the min operation should be used instead of the max operation in *(2b)*.

Chow test [28] is the most commonly used to test for the presence of a structural break in the time series. The model in effect uses an *F*-test to determine whether a single regression is more efficient than two separate regressions with two subsamples.

Suppose that we model the series as

(3) $$\begin{array}{c} y_{t}=\alpha +\beta x_{t}+\varepsilon_{t}. \end{array}$$

If we split the series into two subsamples at the point of structural break, then we have

(3a) $$\begin{array}{c} y_{t}=\alpha_{1}+\beta_{1}x_{t}+\varepsilon_{1t},\;\;y_{t}=\alpha_{2}+\beta_{2}x_{t}+\varepsilon_{2t}. \end{array}$$

The null hypothesis is *α*_1_ = *α*_2_ = *α* and *β*_1_ = *β*_2_ = *β*. If the null hypothesis is accepted then *(3a)* can be expressed as a single regression equation as (3). The assumption here is that errors “*ɛ*” are independent and identically distributed from a normal distribution with unknown variance.

If RSS_c_ is the sum of squared residuals from the whole series, RSS_1_ and RSS_2_ represent the sum of squared residuals from subsample 1 of size *n*_1_ and subsample 2 of size *n*_2_, respectively, and *k* is the total number of parameters, then Chow test statistic is computed as

(3b) $$\begin{array}{c} F\sim \frac{\text{RS}{\text{S}}_{c}-\left(\text{RS}{\text{S}}_{1}+\text{RS}{\text{S}}_{2}\right)/k}{\left(\text{RS}{\text{S}}_{1}+\text{RS}{\text{S}}_{2}\right)/\left(n_{1}+n_{2}-2k\right)}. \end{array}$$

The test statistic in *(3b)* follows *F* distribution with degrees of freedom (*k*, *n*_1_ + *n*_2_ − 2*k*). Finding the critical value from the *F*-test table, conclusion can be made about the null hypothesis.

## 3. Results and Discussion

**Figure 1:**
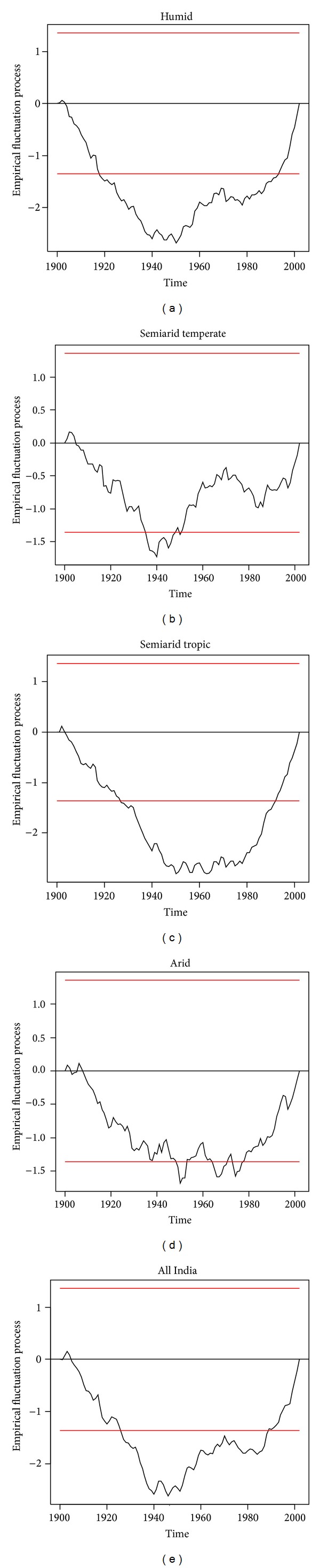
Structural break in annual average temperature.

Empirical fluctuation process according to CUSUM test technique is applied on the time series and pertinent results are shown in Figure 1, and results corresponding to the Chow test statistic (*F* value) and the quantifications corresponding to (1) are reported in Table 2.

Structural changes in annual temperature for all agroclimatic zones and all India are shown in Figures 1(a) to 1(e). A perusal of these indicates that there was a structural change in temperature around 1970 in the humid and semiarid tropical zones. We test the significance of this tentatively observed structural change by applying Chow test and find that the observed structural breaks are highly significant (*P* < 0.001). At the national level, there was significant rise (0.004°C per annum) in temperature during 1901 to 1972, and afterwards it accelerated and increased at a rate of 0.018°C per annum (Table 1).

For the humid zone, the CUSUM test suggests a significant structural break in annual temperature in 1972 which is confirmed by the Chow test. In this zone according to (1) we find a significant rise in temperature at a rate 0.005°C per annum up to 1972 and at an accelerated rate of 0.018°C afterwards. In the semiarid tropic zone we observe 1970 as the point of structural change in annual temperature, and the Chow test confirms it. Between 1901 and 1970 the annual temperature in this zone increased at a rate of 0.004°C and at a rate of 0.020 afterwards.

Based on CUSUM test we tentatively chose 1974 as the point of structural change in temperature in the semiarid temperate and 1971 for the arid zone. The Chow test concludes that these structural breaks persist in these zones. In both zones, the annual temperature had been increasing at similar rates before (0.005°C) and after (0.023°C) the observed structural breaks there.

**Figure 2:**
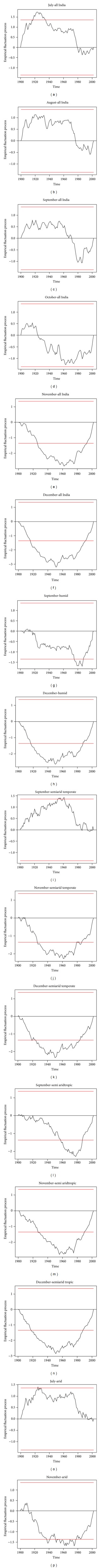
Structural break in monthly average temperature.

We also examined the structural breaks in the monthly mean temperature. We observe multiple structural breaks around 1970 at all India as well as zone level. At the national level, structural changes are noticed in the rainy months of July, August, and September and in the postrainy months of November and December (Figures 2(a) to 2(f)). Considerable structural changes are observed in July, August, and September of 1970. For October the year of structural break is identified as 1967. For November and December structural changes are observed in 1971. The Chow test confirms these changes except for September. The estimated rates of change in monthly temperature are reported in Table 2.

Results of CUSUM test for change in temperature in different agroclimatic zones are shown in Figures 2(g) to 2(p). These figures suggest that, in humid zone, there are considerable changes in September 1969 and December 1970. In semiarid temperate and semiarid tropic zones, the changes are found in the months of September, November, and December. In semiarid temperate zone, this change appears in November 1969, September 1970, and December 1973, while in semiarid tropic zone, the break is observed in September 1967, November 1970, and December 1970. In arid zone, the structural break is found in November 1970 and July 1972. These changes are statistically significant. The findings reveal that there is no significant change in temperature in May-June and January which are considered to be the hottest and coldest months, respectively.

Anthropogenic activities are the main drivers behind climate change. We feel that changes in land use, intensive cropping, deforestation, land clearings, and industrial development have led to a rise in carbon-di-oxide emissions and a structural break in temperature series around 1970. Increased production of goods and services, changes in the production structure, increased transportation, a higher demand for all kinds of consumer goods, and so forth contribute to a higher pressure on the atmosphere there by increasing the greenhouse gas concentration. Green revolution which denotes spectacular increase in production of food grains and other agricultural commodities started in India in the mid-1960s. Following that, there was a significant increase in area under rice, the largest agricultural activity for methane emission in India. Increased area under rice has added significant amount of greenhouse gas emission. This was accompanied by increased use of agrochemicals, diesel, and electricity (Total consumption of fertilizers in India increased from 785 thousand tons in 1965-66 to 2177 thousand tons in 1970-71 and the number of tractors from 54 thousand to 148 thousand. Rice area though did not increase much during this period, but the proportion of rice irrigated increased from 35 % to 40%. Between 1960-61 and 1970-71 the index of industrial production increased from 15.6 to 28.1, electricity generated from 17 to 57 billion KWH, and the consumption of petroleum products from 7.7 to 17.9 million tons [29]. These trends indicate an increase in greenhouse gas emission leading to a structural change in temperature around 1970.) [30] that also contributed to carbon-di-oxide emission.

Another notable factor that has contributed to rise in temperature has the significant increase in ruminant population, the second largest agricultural enterprises contributing to greenhouse gas emission after rice. Land-cover and land-use (LCLU) changes have significant climate impacts in tropical coastal regions with the added complexity of occurring within the context of a warming climate [31]. LCLU changes produced the largest near-surface air temperature differences over heavily urbanized regions [31].

## 4. Conclusion

This study has estimated structural changes in annual and mean monthly temperature at all India and regional levels during the period 1901–2001. A structural break in annual temperature occurred around 1970 at the national and regional levels because of significant increases in the industrial and agricultural activities. We observed structural breaks in mean monthly temperatures in the rainy (July–September) and postrainy months (October–December) around the same time, indicating that it was the rise in temperature during these periods that caused structural breaks in annual temperature. Interestingly, there was no significant change in temperature in May-June and January which are considered to be the hottest and coldest months, respectively. These findings have implications for efforts and investments in adaptation and mitigation strategies to cope with global warming especially in the agricultural sector which is more sensitive to climate change.

## Figures and Tables

**Table 1:** Breaks in annual average temperature.

Region	All India	Humid	Semiarid temperate	Semiarid tropic	Arid
Break	1972	1972	1974	1970	1971
Rate of change in temperature from 1901 to break point	0.004	0.005	0.005	0.004	0.005
Rate of change in temperature from break point to 2002	0.018	0.018	0.023	0.020	0.023

**Table 2:** Structural break point alongwith their significance level by Chow test.

Month	Zone	Break	Chow statistic	Rate of change in temperature from 1901 to break point	Rate of change in temperature after break point
Annual average temperature	All India	1972	14.547∗∗∗	0.004∗∗∗	0.014∗∗∗
Annual average temperature	Humid	1972	19.565∗∗∗	0.005∗∗∗	0.013∗∗∗
Annual average temperature	Semiarid temperate	1974	1.223	0.005∗∗	0.018∗∗
Annual average temperature	Semiarid temperate	1970	40.316∗∗∗	0.004∗∗	0.016∗∗∗
Annual average temperature	Arid	1971	1.231	0.005∗∗	0.018∗∗
July	All India	1970	4.269∗∗∗	−0.007∗∗∗	0.023∗∗∗
August	All India	1970	3.192∗∗∗	−0.003∗∗	0.02∗∗∗
September	All India	1970	0.063	−0.003∗	0.024∗∗∗
October	All India	1967	5.420∗∗	−0.003	0.016∗∗
November	All India	1971	41.126∗∗∗	0.007∗∗	0.030∗∗∗
December	All India	1971	37.215∗∗∗	0.012∗∗∗	0.017∗∗
September	Humid	1969	2.845∗	0.001	0.019∗∗∗
December	Humid	1970	20.310∗∗∗	0.014∗∗∗	0.022∗∗
September	Semiarid temperate	1970	5.204∗∗	−0.005	0.021∗∗
November	Semiarid temperate	1969	18.198∗∗∗	0.010∗∗	0.027∗
December	Semiarid temperate	1973	16.050∗∗∗	0.013∗∗∗	0.021
September	Semiarid tropic	1967	21.538∗∗∗	−0.005∗∗	0.024∗∗∗
November	Semiarid tropic	1970	35.158∗∗∗	0.009∗∗	0.032∗∗
December	Semiarid tropic	1970	28.886∗∗∗	0.013∗∗∗	0.021∗∗
July	Arid	1972	6.593∗∗	−0.008	0.039∗∗
November	Arid	1970	10.599∗∗∗	0.005	0.033∗∗

∗∗∗Significant at 1% level of significance.

∗∗Significant at 5% level of significance.

∗Significant at 10% level of significance.
